# A Rare Case of Metastatic Breast Cancer in the Bladder

**DOI:** 10.7759/cureus.92232

**Published:** 2025-09-13

**Authors:** Lydia MY Chang, Parthvi Vanalia, Adam Jones

**Affiliations:** 1 Core Surgical Training, Health Education England North West Deanery, Northwest, GBR; 2 Urology, Lancashire Teaching Hospitals National Health Service (NHS) Trust, Preston, GBR; 3 Urology, Health Education England North West Deanery, Northwest, GBR

**Keywords:** : acute kidney injury, bilateral hydroureteronephrosis, bladder ca, metastatic breast cancer (mbc), rare metastasis

## Abstract

This case describes the rare occurrence of breast cancer with metastasis to the bladder. This woman in her 60s, was first diagnosed with breast cancer in the mid-2000s. Nearly 20 years later, she developed recurrence at her primary site and metastasis to the skin and bones, and subsequently to the bladder. The involvement of the bladder initially presented as a stage 3 acute kidney injury during routine blood tests whilst the patient was undergoing chemotherapy for local breast cancer recurrence and metastasis. Initial ultrasound imaging showed bilateral hydroureteronephrosis. Subsequent computed tomography (CT) scan with contrast revealed a thickened bladder wall in addition to the bilateral hydroureteronephrosis. She underwent bilateral nephrostomy insertions under local anaesthesia as an emergency, and a flexible cystoscopy, which revealed a tumour in the bladder. This patient then underwent a transurethral resection of bladder tumour (TURBT) and histology samples from this resection revealed metastatic breast cancer in the bladder rather than a primary tumour from the bladder.

## Introduction

Breast cancer is the most common cancer in the United Kingdom, making up 15% of new cancer diagnoses every year [1]. Globally, it remains the most frequently diagnosed malignancy among women and a leading cause of cancer-related mortality [2]. While advances in screening and systemic therapies have improved survival, metastatic breast cancer is not uncommon, with literature citing nearly 30% of women who were initially diagnosed with early-stage breast cancer will eventually develop metastases [3].

Breast cancers commonly metastasise to the brain, lung, liver and bones [4]. Metastasis of breast cancer to the bladder is an extremely rare occurrence, with less than a 100 cases reported to date since its first documentation, mostly reported as isolated case reports or small case series [5,6]. Bladder metastasis is often clinically unsuspected, and patients may initially present with non-specific lower urinary tract symptoms such as frequency, urgency or haematuria, whilst others are asymptomatic until obstructive uropathy or acute kidney injury (AKI) develops [7].

Given its rarity, each additional case contributes valuable insight into the spectrum of presentations, diagnostic pitfalls, and management strategies. As in our case, it was detected incidentally during evaluation of renal dysfunction. This report highlights a case of metastatic breast cancer to the bladder presenting with AKI, underscoring the importance of maintaining clinical suspicion and utilising histopathological confirmation in patients with a known history of breast malignancy.

## Case presentation

This patient, a woman in her 60s, who was a lifelong smoker of around 20 pack years, was initially diagnosed with left breast invasive lobular carcinoma nearly 20 years ago in her country of origin. She underwent a wide local excision and received adjuvant chemotherapy, adjuvant radiotherapy (presumably breast only) and hormone therapy. 

Three years ago, she developed recurrence in the left breast - a multifocal tumour - and underwent left mastectomy and axillary exploration with negative lymph node involvement. She then moved to the United Kingdom and was started on adjuvant chemotherapy (Paclitaxel); however, this was discontinued after her ninth dose as she developed acute heart failure and was hospitalised for the same. She recovered from this acute episode and as repeated staging scans showed no progression and stable disease, she was discharged from the oncology service. 

**Figure 1:**
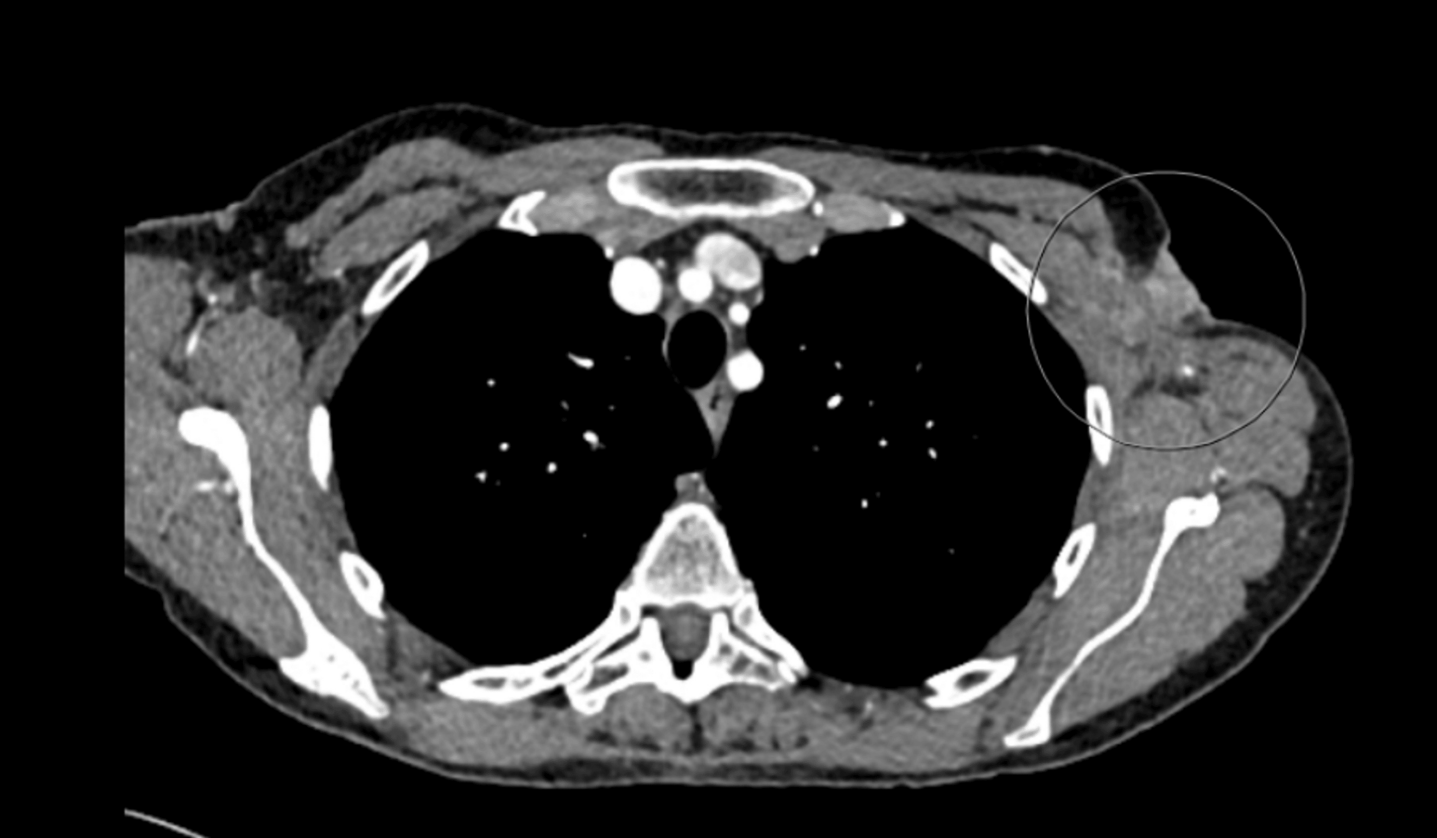
Left axilla soft tissue local recurrence and subcutaneous nodules of left chest wall

**Figure 2:**
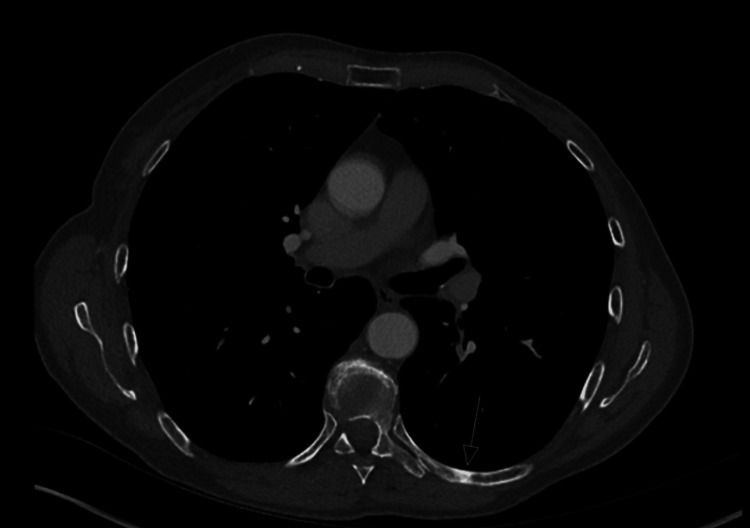
Left posterior 8th rib sclerotic lesion axial view

**Figure 3:**
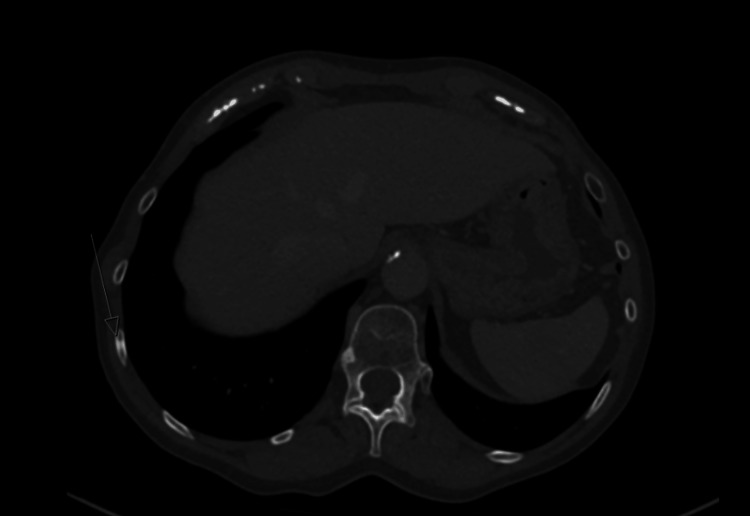
Right 9th rib sclerotic lesion

**Figure 4:**
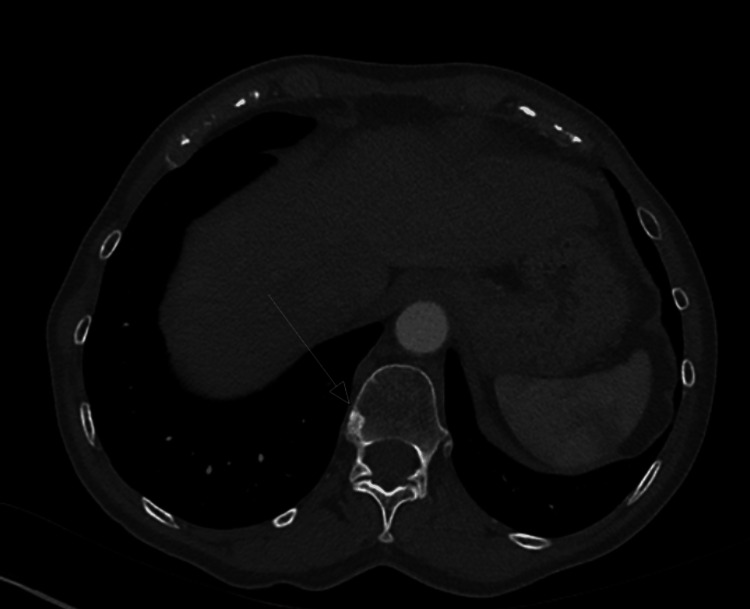
T12 vertebral body sclerotic lesion axial view

**Figure 5:**
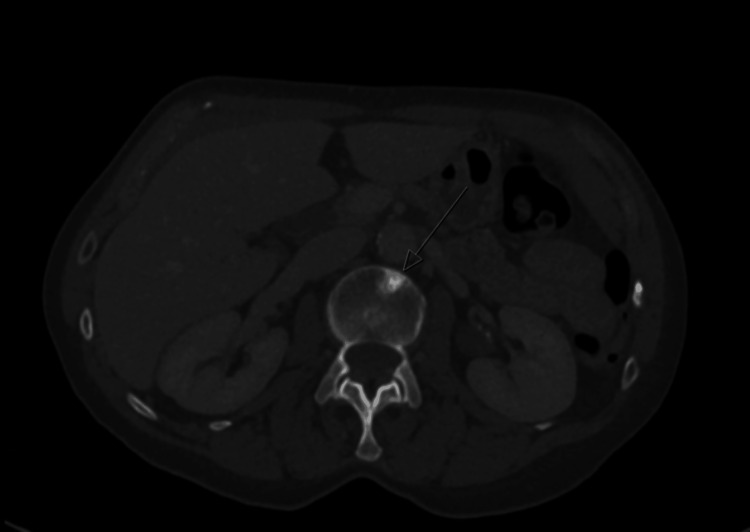
L2 vertebral body sclerotic lesion axial view

A few months later, she developed a lump in her left axilla and skin nodules on the left chest wall. Repeated CT scans of the thorax abdomen and pelvis (CT TAP) showed local recurrence in the left axilla, subcutaneous nodules of the left chest wall (Figure 1) and bony metastases to multiple ribs (Figures 2, 3) and vertebral bodies (Figures 4, 5).

She once again underwent chemotherapy (Capecitabine). Subsequent CT scan showed interval disease progression and she was started on another chemotherapy agent (Vinorelbine) and underwent electrochemotherapy (Bleomycin) to the skin nodules.

**Figure 6:**
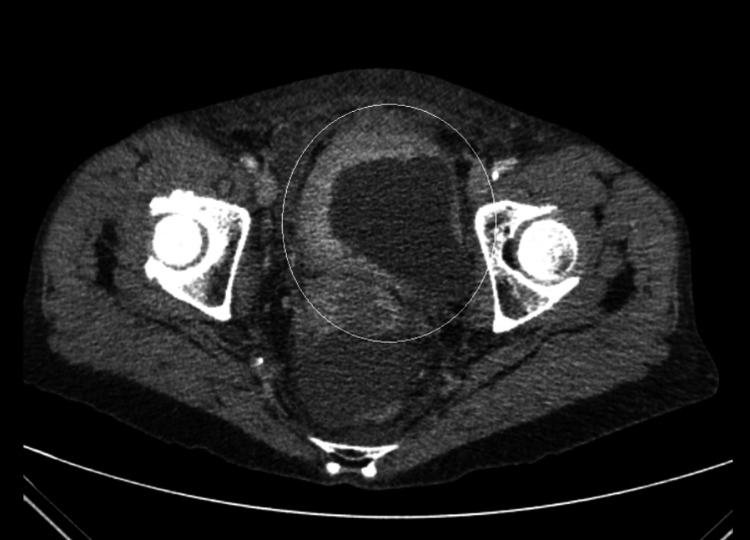
Enhancing thickening in bladder wall

**Figure 7:**
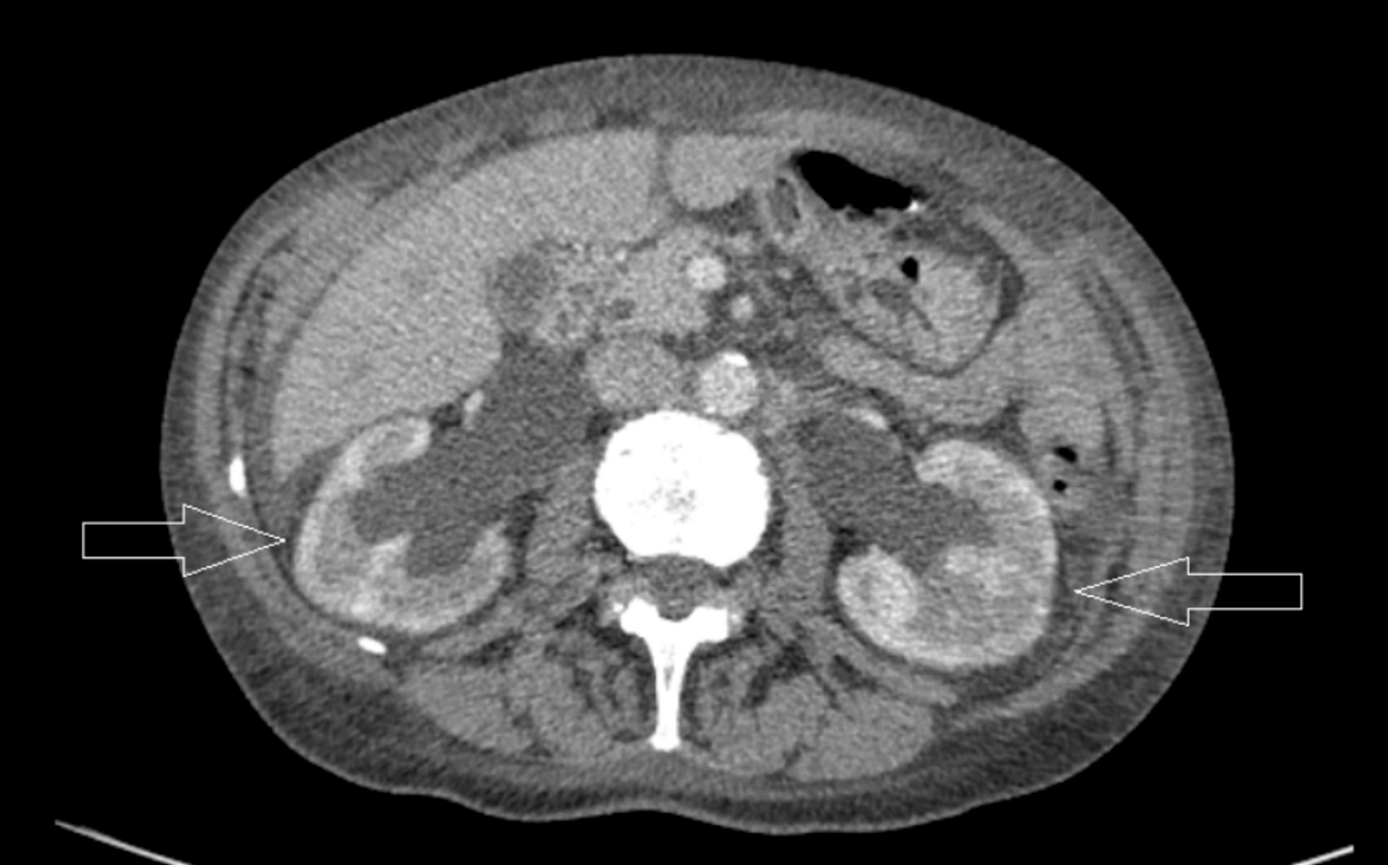
Bilateral hydroureteronephrosis

One year post initial diagnoses of recurrence and metastasis, her routine blood tests showed an AKI 3 with an increase in creatinine level to 218 μmol/L from 78 μmol/L (reference range 45-84 μmol/L), and estimated glomerular filtration rate (eGFR) decreasing to 22 from 69 (reference range eGFR>90). She was admitted under the medical team and as her AKI was not responsive to fluids, they performed an ultrasound of the kidneys, ureter and bladder, which revealed bilateral moderate hydronephrosis and proximal hydroureters. Her repeated CT TAP scan showed an enhanced thickening of the bladder wall (Figure 6) in addition to bilateral moderate hydroureteronephrosis (Figure 7). 

She was then referred to the urology service and had bilateral nephrostomies inserted. Her AKI improved post nephrostomies insertion. At the time of presentation to urology, she denied any lower urinary tract symptoms such as frequency, urgency, dysuria, nocturia or haematuria. There were also no records of recurrent urinary tract infections. Bimanual examination under anaesthesia revealed a fixed bladder with a hard palpable mass. She had a flexible cystoscopy performed, which showed irregular and thick bladder changes on the posterior wall and right lateral wall with raised spots of necrosis, findings suggestive of primary muscle-invasive bladder cancer. She subsequently had telescopic urethral resection of bladder tumour (TURBT) and histology examination showed tumour cells that are strongly and diffusely positive with mammaglobin, GATA3 and CK7. There is focal but strong positivity with gross cystic disease fluid protein-15 (GCDFP-15). It tested negative for markers CK20, E-cadherin, p63, p40 and thrombomodulin. Oestrogen and progesterone receptors were also negative. The histological appearances and immunoprofile are those of metastatic lobular carcinoma of the breast and confirmed bladder tumour from breast cancer metastasis as opposed to a tumour of primary urothelial origin. She was then commenced on oral chemotherapy (Vinorelbine) but unfortunately passed away a few months after the bladder metastases diagnosis. 

## Discussion

Metastasis to the bladder from a primary tumour of breast origin is extremely rare. Breast cancer commonly metastasises to the bone, brain, liver and lung. Bladder metastases are uncommon and only accounts for <2% of bladder tumours [8]. 

There are two main types of breast carcinoma, mainly invasive lobular carcinoma (ILC) and invasive ductal carcinoma (IDC). IDC is more common than ILC, which only accounts for 5%-15% of breast cancers; however, there is a higher propensity for ILC to metastasise to uncommon sites such as the bladder. This is due to the fact that ILC tends to spread to serosal surfaces, which accounts for the majority of breast carcinoma metastasis to the bladder [7]. It is also reported in a study that roughly one-third of bladder metastases are from ILC [5]. Interestingly, a separate study reported that IDC was slightly more common than ILC when metastasing to the bladder [6]. In the same study, they have also found that the median time from primary breast cancer diagnosis to bladder metastasis was around 5.6 years, but intervals of up to 28 years were also reported.

Differentiating primary bladder carcinoma from metastases can be challenging, and immunohistochemical staining plays a vital role in distinguishing between the two [7]. Markers such as estrogen receptor (ER), progesterone receptor (PR), human epidermal growth factor receptor 2 (HER2), GCDFP-15 and mammaglobin are commonly used to identify tumours of breast origin [9]. 

Treatment of breast cancer metastases remains largely palliative, and focuses on systemic treatment, which includes chemotherapy, hormone therapy or targeted therapy, guided by the receptor status of the tumour. Local treatments such as TURBT are mainly symptomatic treatment only, such as to reduce incidences of haematuria, or to relieve obstruction [8]. The prognosis of bladder metastasis is generally poor, with median survival rates of nine to 18 months from diagnosis of bladder metastasis [6].

## Conclusions

Bladder metastasis from primary breast carcinoma is extremely rare. This case underscores the clinical challenge of diagnosing bladder metastasis from breast cancer, an entity so rare that it is often not considered in the initial differential diagnosis. Our patient presented with AKI and bilateral hydroureteronephrosis but without lower urinary tract symptoms. CT imaging revealed enhancing thickening of bladder wall, mimicking primary bladder carcinoma. Only histopathological examination with immunohistochemical staining confirmed the diagnosis of metastatic lobular breast carcinoma.

The broader implication of this case is the need for heightened index of suspicion amongst physicians especially in patients with a prior history of breast cancer or any other malignancies, presenting with new onset lower urinary tract symptoms, recurrent urinary tract infections or AKI. Whilst AKI is a known side effect of various chemotherapy agents, this case highlights the importance of an initial ultrasound sonography scan in patients whose AKI does not improve with intravenous fluid rehydration. Imaging and cystoscopy may be inconclusive, and ultimately tissue sampling is critical to aid in diagnosis. Differentiating primary bladder tumour from bladder metastasis can be challenging and immunohistochemistry analysis is a key factor in distinguishing between both. Moreover, this case highlights that bladder metastases, although rare, can present decades after initial diagnosis, reflecting the long natural history of breast cancer recurrence.
